# *In vivo* and *in silico* Virulence Analysis of *Leptospira* Species Isolated From Environments and Rodents in Leptospirosis Outbreak Areas in Malaysia

**DOI:** 10.3389/fmicb.2021.753328

**Published:** 2021-11-05

**Authors:** Noraini Philip, Jaeyres Jani, Nurul Natasya Azhari, Zamberi Sekawi, Vasantha Kumari Neela

**Affiliations:** ^1^Department of Medical Microbiology, Faculty of Medicine and Health Sciences, Universiti Putra Malaysia, Selangor, Malaysia; ^2^Borneo Medical and Health Research Center, Universiti Malaysia Sabah, Sabah, Malaysia

**Keywords:** *Leptospira*, virulence, *in vivo*, *in silico*, hamster, genome

## Abstract

The zoonotic disease leptospirosis is caused by pathogenic species of the genus *Leptospira*. With the advancement of studies in leptospirosis, several new species are being reported. It has always been a query, whether *Leptospira* species, serovars, and strains isolated from different geographical locations contribute to the difference in the disease presentations and severity. In an epidemiological surveillance study performed in Malaysia, we isolated seven novel intermediate and saprophytic species (*Leptospira semungkisens*is, *Leptospira fletcheri*, *Leptospira langatensis*, *Leptospira selangorensis*, *Leptospira jelokensis*, *Leptospira perdikensis*, *Leptospira congkakensis*) from environments and three pathogenic species from rodents (*Leptospira borgpetersenii* strain HP364, *Leptospira weilii* strain SC295, *Leptospira interrogans* strain HP358) trapped in human leptospirosis outbreak premises. To evaluate the pathogenic potential of these isolates, we performed an *in vivo* and *in silico* virulence analysis. Environmental isolates and strain HP364 did not induce any clinical manifestations in hamsters. Strain SC295 caused inactivity and weight loss with histopathological changes in kidneys, however, all hamsters survived until the end of the experiment. Strain HP358 showed a high virulent phenotype as all infected hamsters died or were moribund within 7 days postinfection. Lungs, liver, and kidneys showed pathological changes with hemorrhage as the main presentation. *In silico* analysis elucidated the genome size of strain HP358 to be larger than strains HP364 and SC295 and containing virulence genes reported in *Leptospira* species and a high number of specific putative virulence factors. In conclusion, *L. interrogans* strain HP358 was highly pathogenic with fatal outcome. The constituent of *Leptospira* genomes may determine the level of disease severity and that needs further investigations.

## Introduction

Leptospirosis is a zoonotic disease, with rodents being the main transmitting source. Humans get infected either by direct contact with the animals or indirectly from environments contaminated with animal urine. There are three classes of *Leptospira*: nonpathogenic, intermediate, and pathogenic. To date, there have been 66 *Leptospira* species (Masuzawa et al., 2018; Thibeaux et al., 2018; Vincent et al., 2019; Casanovas-Massana et al., 2020) and more than 300 serovars identified. The majority of the non-pathogens and intermediate species come from environments (soil and water), while the pathogens are excreted in the urine of reservoir animals (Levett, 2001; Putz and Nally, 2020). The pathogenic *Leptospira* species, serovars, and strains are known to cause leptospirosis and contribute to the broad spectrum of the disease presentations (Vinetz, 2001). The pathogenic potential of *Leptospira* species has been attributed to the presence of genes that encode the virulence phenotype (Fouts et al., 2016). There are unique genetic determinants that influence the pathogenic *Leptospira* to adhere, invade, disseminate, escape from host defense, and establish systemic infection. These genetic elements may be absent or less expressed in intermediate and nonpathogenic *Leptospira*. When compared to several other bacteria, pathogenic *Leptospira* species do not have any typical exotoxin-encoding genes (Ren et al., 2003; Nascimento et al., 2004). Similarly, the lipopolysaccharide in pathogenic *Leptospira interrogans* has been reported to have lower endotoxic activities than other bacteria (Isogai et al., 1986, 1989; Vinh et al., 1989; Bulach et al., 2000; Adler, 2015). Endostatin-like outer membrane proteins, such as LipL32/53, Lsa21/32/63, LenA, LigA, and LigB (Choy et al., 2007; Stevenson et al., 2007; Atzingen et al., 2008; Hauk et al., 2008; Oliveira et al., 2010; Vieira et al., 2010; Domingos et al., 2015), have been identified to participate in the adherence of pathogenic *Leptospira* to host cells by binding to fibronectin, laminin, and collagens of extracellular matrix, whereas collagenase (Kassegne et al., 2014), *invA* (Fraser and Brown, 2017), M16 type of metalloprotease/metallopeptidase (Ren et al., 2003), mammalian cell entry protein, and endoflagellum of pathogenic *Leptospira* have been reported to contribute to leptospiral invasiveness (Liao et al., 2009; Zhang et al., 2012). The hemolysins (sphingomyelinase-type hemolysins, sphH)—SphH pore-forming protein Sph1-3, HlpA, TlyA, and Loa22—are recognized as the strong inducer of interleukin 1β (IL-1β), IL-6, and tumor necrosis factor α production (Lee et al., 2002; Ristow et al., 2007; Wang et al., 2012; Hsu et al., 2021). The vWA proteins encoded by genes *vwa-I* and *vwa-II* have been shown to induce hemorrhage in *L. interrogans* (Fang et al., 2018). It is believed that there could be more virulent determinants that are yet to be discovered.

With the advancement in genomics, rise in leptospirosis cases, and studies from various geographical locations, more new species and serovars are being reported. It is vital to identify and characterize the circulating species and serovars in every country to understand the epidemiology and pathogenic potential to combat the illness. In an earlier study, we reported seven novel species classified as nonpathogenic isolated from soil and water samples from leptospirosis outbreak areas in Malaysia (Vincent et al., 2019). We also isolated a few pathogenic species such as *L. interrogans*, *Leptospira kirschneri*, *Leptospira weilii*, and *Leptospira borgpetersenii* from rodents captured in the same areas (Azhari et al., 2018). In a very recent study, we reported *L. interrogans* and *L. kirschneri* as the human-infecting *Leptospira* species in Malaysia (Philip et al., 2020). In the present study, we aimed to evaluate the *in vivo* virulence of the pathogenic (from rodents) and novel *Leptospira* species (from environments) in a hamster model and to perform a comparative genomics study to elucidate the virulence characteristics of *Leptospira* species and identify potential virulence factors.

## Materials and Methods

### Animal Experiments and Ethics Approval

Experiments were conducted following the guidelines of the Code of Practice for the Care and Use of Animals for Scientific Purposes, Universiti Putra Malaysia. Male golden Syrian hamsters purchased from Monash Universiti Malaysia, Bandar Sunway, Selangor, aged between 4 and 6 weeks, were housed individually in cages with sterile sawdust bedding. The animals were fed with their routine food, watered in sterile bottles throughout the study, and were acclimatized for 7 days before infection. All animal procedures carried out in this study were reviewed and approved by the Institutional Animal Care and Use Committee (IACUC), Universiti Putra Malaysia with Animal Use Protocol (AUP) number UPM/IACUC/AUP-R044/2018.

### Infection, Monitoring, and Euthanasia of Animals

Upon completion of 7 days of acclimatization, the hamsters were intraperitoneally (IP) injected with 2 × 10^8^ (Matsui et al., 2011) of second passages of leptospires cultures from environmental and animal isolates (Table 1) in 500 μL Ellinghausen, McCullough, Johnson and Harris (EMJH) medium. Control animals were injected (IP) with 500 μL sterile EMJH medium. Four animals were infected with leptospires in each *Leptospira* species and control. Hamsters injected with environmental isolates were monitored for 14 days, whereas for pathogenic isolates up to 21 days. Those animals that were moribund during the course of study characterized by significant weight loss ≥ 10%, lethargy, gait difficulty, dyspnea, and prostration were anesthetized with 100 mg/kg ketamine and 5 mg/kg xylazine IP injection and euthanized by atlanto-occipital dislocation and dissected after cardiac puncture. All animals that survived until the end of the study were euthanized on days 14 (environmental) and 21 (animal isolates) postinfection (p.i.). Prior to euthanization, blood was collected by cardiac puncture for direct culture in the EMJH medium and for leptospiral DNA detection in the EDTA tube. Approximately 25 mg of lungs, liver, and kidneys were collected in absolute ethanol for leptospiral DNA detection. The remaining part of the organs was preserved in neutral-buffered formalin for histopathology observation.

**TABLE 1:** List of *Leptospira* species used in *in vivo* study.

No.	Species	Strain	Source	References
1	*L. borgpetersenii*	HP364	Rodents	Azhari et al., 2018
2	*L. weilii*	SC295	Rodents	Azhari et al., 2018
3	*L. interrogans*	HP358	Rodents	Azhari et al., 2018
4	*L. semungkisensis*	SSS9	Environments (soil)	Vincent et al., 2019
5	*L. fletcheri*	SSW15	Environments (water)	Vincent et al., 2019
6	*L. congkakensis*	SCS9	Environments (soil)	Vincent et al., 2019
7	*L. jelokensis*	L5S1	Environments (soil)	Vincent et al., 2019
8	*L. perdikensis*	HP2	Environments (water)	Vincent et al., 2019
9	*L. langatensis*	SSW18	Environments (water)	Vincent et al., 2019
10	*L. selangorensis*	SSW17	Environments (water)	Vincent et al., 2019

### Detection of Leptospires in Blood and Organs

DNA from 200 μL of blood and 25 mg of organs was extracted using the DNeasy blood and Tissue kit (Qiagen, Germany) following the manufacturer’s instructions. Detection of *lipL32* gene (Stoddard et al., 2009) was performed using the quantitative polymerase chain reaction (PCR) platform on Eppendorf MasterCycler^®^ Realplex. Final reaction for the real-time PCR contained 12.5 μL Quantinova probe PCR kits (2 × concentration), 1 μL of 10 pmol of each primer, 0.5 μL of 10-pmol probe, 5 μL of RNase free water, and 5 μL of DNA extracted from blood or tissue samples in a final volume of 25 μL. The amplification protocol started with 8 min at 95°C, followed by 45 cycles of 95°C for 3 s and 58°C for 15 s, and finished with a cool cycle at 45°C for 90 s. Extracted DNA from a pure culture of *L. interrogans* strain HP358 and RNase free water were used as positive and negative controls.

### Observation of Tissue Damages in the Infected Animals by Histopathology

For each organ, formalin-fixed paraffin-embedded tissue blocks were subjected to hematoxylin and eosin (H&E) stain. Formalin-fixed tissues were embedded in paraffin, cut into thin sections (∼4 mm), and stained with H&E by standard protocol. Tissue damages are recorded as per previously reported criteria (Marinho et al., 2009; Matsui et al., 2011; Villanueva et al., 2014).

### Genome Sequencing, *de novo* Assembly, Phylogenetic Tree, and Annotation

The genomic DNA of *L. borgpetersenii* strain HP364, *L. weilii* strain SC295, and *L. interrogans* strain HP358 was extracted from pure cultures using DNeasy Blood and Tissue kits (Qiagen) according to the manufacturer’s instruction. The DNA library for whole-genome sequencing was prepared using the IGP-NGSP Illumina Library and subsequently sequenced through the Miseq instruments platform. The quality of raw reads of the sequenced genomes was checked using FastQC and preprocessed using BBMap version 38.43 tool (Bushnell et al., 2017). The adapters and reads with less than 50 bp were trimmed based on the phred quality score (below Q30) using BBduk.sh. To avoid bias using reference mapping, *de novo* genome assembly was performed for all the three species using SPAdes version 3.11.1 (Bankevich et al., 2012).

Single-nucleotide polymorphism (SNP)–based phylogenetic tree was performed for the three pathogenic *Leptospira* strains described previously (*L. borgpetersenii* strain HP364, *L. weilii* strain SC295, and *L. interrogans* strain HP358) and another 49 *Leptospira* strains (including the seven *Leptospira* species from environment isolates used in this study) (Supplementary File 1, Table 1). The whole-genome sequences of the 49 strains were extracted from NCBI GenBank. The core SNP was determined using the kSNP3 package (Gardner et al., 2015). All SNP matrices were aligned using CLUSTALW, and SNP-based phylogenetic tree was performed using the maximum likelihood method in MEGAX (Molecular Evolutionary Genetic Analysis) software (Kumar et al., 2018). The significance of the branching patterns was evaluated through bootstrap analysis of 1,000 replicates.

The generated contigs of *L. borgpetersenii* strain HP364, *L. weilii* strain SC295, and *L. interrogans* strain HP358 were annotated using Rapid Annotation using Subsystem Technology (RAST) server version 2.0 (Aziz et al., 2008). Gene and putative protein-coding sequences of *L. borgpetersenii* strain HP364, *L. weilii* strain SC295, and *L. interrogans* strain HP358 were predicted with the GeneMark program (Besemer et al., 2001).

### Pan-Genome Analysis

A total of 30 genomes of *Leptospira* strains (Supplementary File 1, Table 2) were subjected to pan-genome analysis using Roary V3.11.2 (Page et al., 2015). The input files used were the GFF3 (General Feature Format version 3) format generated from annotated assembly (GeneMark) (*L. borgpetersenii* strain HP364, *L. weilii* strain SC295, *L. interrogans* strain HP358) and GenBank files obtained from the NCBI website (27 strains). For definition of core genes in all strains, the threshold of 99% was used, whereas for sequence comparison between strains, a user-defined percentage sequence identity (default 95%) was performed with BLASTP (Sitto and Battistuzzi, 2020). Using conserved gene neighborhood information, homologous groups containing paralogs were split into groups of true orthologs. The strains were clustered based on gene presence in the accessory genome, weighted by the total and shared genomes. A core-genome phylogenetic tree was constructed using the maximum likelihood method in MEGAX with a bootstrap analysis of 1,000 replicates. The list genes of the three pathogenic species (*L. borgpetersenii* strain HP364, *L. weilii* strain SC295, and *L. interrogans* strain HP358) were extracted from the pan-genome data for comparison of core and specific gene.

### Virulence Factor Analysis

A list of known virulence factors in *Leptospira* species was generated from literature reviews (Murray, 2015; Gomes-Solecki et al., 2017; Picardeau, 2017). The amino acid sequences of these virulence factors were retrieved from UniProt database and were subjected to a BLAST search against the genome of *Leptospira* species used in the *in vivo* study and 29 representatives *Leptospira* genomes using local blast (tblastn) in BlastSation software in the following link.^1^ Additional five virulence factors with nucleotide sequences were retrieved from NCBI GenBank database and blasted against the genomes of *Leptospira* using homology blastn method (Altschul et al., 1990). Besides the known virulence factors in *Leptospira* genome, potential virulence factors from other bacterial species were also investigated in *L. borgpetersenii* strain HP364, *L. weilii* strain SC295, and *L. interrogans* HP358 through ortholog gene identification present in full datasets of virulence factor database (VFDB) (Chen et al., 2016) and Victors database^2^ (Sayers et al., 2019) with criteria of more than 70% alignment coverage (bit score) and 40% identity (Rost, 1999) using Blastation software.

## Results

### Clinical Response to Infection

**FIGURE 1:**
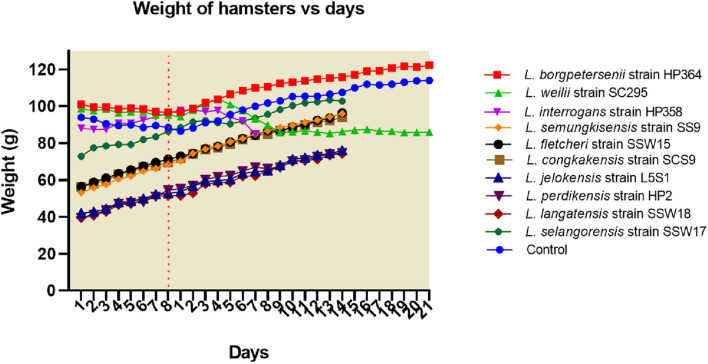
Weight of hamsters. Only hamsters infected with *L. weilii* strain SC295 and *L. interrogans* strain HP358 showed weight loss after infections. The dot line (day 8) represents the day in which the hamsters were infected with leptospires. Days 1–8 and 1–21 were the acclimatization and postinfection periods, respectively.

**FIGURE 2:**
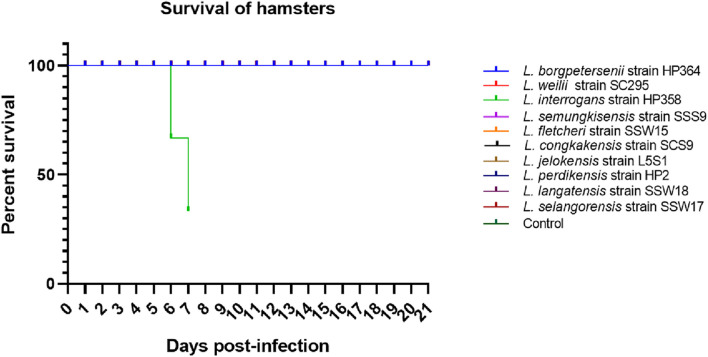
Survival curve. Only *L. interrogans* strain HP358 caused fatality in infected animals.

All hamsters infected with *Leptospira* isolates from environments survived, increased in body weight (Figure 1), and showed no clinical presentations until the animals were euthanized on day 14 (Table 2). Similar to the environment isolates, *L. borgpetersenii* strain HP364 isolated from rodents also did not show any clinical manifestations in hamsters, and all survived until they were euthanized (21st day). Although the hamsters infected with *L. weilii* strain SC295 survived until the end of the study, the animals showed mild weight loss and were less active between day 5 and 9 p.i. On the contrary, *L. interrogans* strain HP358 induced a fatal outcome in infected hamsters. The animals showed loss of appetite and presence of eyes suffusion, were less active, and had difficulty in breathing from day 5 p.i. onward. Two animals died on day 6 and one on day 7 p.i., whereas the remaining one animal was moribund, hence euthanized on day 7 (Figure 2: survival curve).

**TABLE 2:** Clinical manifestations and survival of hamsters infected with *Leptospira* isolates.

No.	Species	Source	Clinical manifestation	Survival
1.	*L. borgpetersenii* strain HP364	Rodents	No	Yes (21 days)
2.	*L. weilii* strain SC295	Rodents	Yes—inactive, weight loss	Yes (21 days)
3.	*L. interrogans* strain HP358	Rodents	Yes—inactive, weight loss, eyes suffusion, hunched back, breathing problem	Two died on day 6, one on day 7, and one moribund animal died on day 7
4.	*L. semungkisensis* strain SSS9	Environments (soil)	No	Yes (14 days)
5.	*L. fletcheri* strain SSW15	Environments (water)	No	Yes (14 days)
6.	*L. congkakensis* strain SCS9	Environments (soil)	No	Yes (14 days)
7.	*L. jelokensis* strain L5S1	Environments (soil)	No	Yes (14 days)
8.	*L. perdikensis* strain HP2	Environments (water)	No	Yes (14 days)
9.	*L. langatensis* strain SSW18	Environments (water)	No	Yes (14 days)
10.	*L. selangorensis* strain SSW17	Environments (water)	No	Yes (14 days)

### Macroscopic and Microscopic Observation of Lungs, Liver, and Kidneys Infected With *Leptospira* Species

**FIGURE 3:**
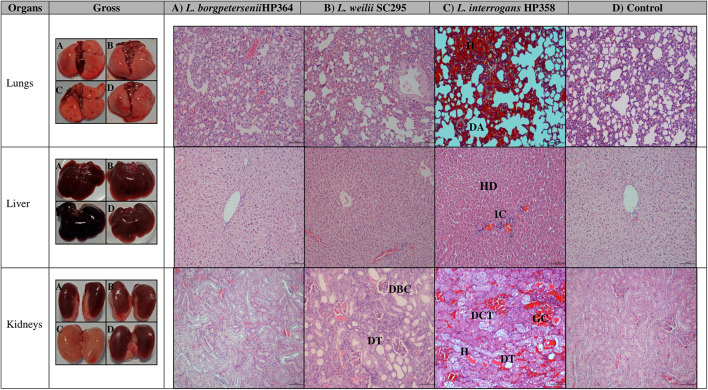
Gross and microscopic observation of organs in infected and control hamsters. H, hemorrhage; DA, dilated alveoli; HD, hepatic disarray; IC, infiltration on inflammatory cells; DBC, dilated Bowman capsule; DT, dilated tubule; DCT, disarrangement of cells in tubules; DT, dilated tubule; GC, glomerulus congestion. Magnification × 100, bar: 100 μm.

Macroscopically, the lungs, liver, and kidneys of hamsters infected with environmental isolates and *L. borgpetersenii* strain HP364 showed normal morphology. Animals infected with *L. weilii* strain SC295 presented normal lungs and liver, whereas the kidneys appeared shrunken. *L. interrogans* strain HP358 induced changes in the lungs, liver, and kidneys of the infected hamsters. The changes included hemorrhage in the lungs, congestion in the liver, and paleness of kidneys (Figure 3).

For microscopic analysis, organs (lungs, liver, and kidneys) from control and infected animals proceeded for H&E staining (Figure 3). Animals infected with *L. borgpetersenii* strain HP364 showed no pathological changes in lungs, liver, and kidneys. In *L. weilii* strain SC295–infected animals, lungs and liver appeared normal, but in kidneys, the tubules and Bowman capsule were dilated. All three organs in hamsters infected with *L. interrogans* strain HP358 showed severe damage. Lungs presented with congestion, hemorrhage, and mild dilation of alveoli, whereas in the liver, hepatic chord with ballooning of hepatocyte was observed. In kidneys, congestion and tubular hemorrhage, mild dilation of tubules, and derangement of epithelial cells were observed.

### Recovery and Detection of Leptospires in Blood and Organs

Leptospires were not detected in hamsters infected with environmental isolates and *L. borgpetersenii* strain HP364 (Table 3). For *L. weilii* strain SC295, leptospires were recovered and detected in kidneys and none from blood or other organs. In *L. interrogans* strain–infected animals, *Leptospira* was recovered and detected in blood, lungs, liver, and kidneys. The leptospiral load was high in all organs in hamsters infected with *L. interrogans* strain HP358 (Table 4).

**TABLE 3:** Recovery and detection of leptospires in blood and organs.

No.	Species	Culture		PCR			
		Blood	Kidneys	Blood	Lungs	Liver	Kidneys
1.	Control	0/4	0/4	0/4	0/4	0/4	0/4
2.	*L. borgpetersenii* strain HP364	0/4	0/4	0/4	0/4	0/4	0/4
3.	*L. weilii* strain SC295	0/4	4/4	0/4	0/4	0/4	4/4
4.	*L. interrogans* strain HP358	1/4*	4/4	1/4*	4/4	4/4	4/4
5.	*L. semungkisensis* strain SSS9	0/4	0/4	0/4	0/4	0/4	0/4
6.	*L. fletcheri* strain SSW15	0/4	0/4	0/4	0/4	0/4	0/4
7.	*L. congkakensis* strain SCS9	0/4	0/4	0/4	0/4	0/4	0/4
8.	*L. jelokensis* strain L5S1	0/4	0/4	0/4	0/4	0/4	0/4
9.	*L. perdikensis* strain HP2	0/4	0/4	0/4	0/4	0/4	0/4
10.	*L. langatensis* strain SSW18	0/4	0/4	0/4	0/4	0/4	0/4
11.	*L. selangorensis* strain SSW17	0/4	0/4	0/4	0/4	0/4	0/4

*For blood samples, blood was not available for the dead animals (3/4).

**TABLE 4:** The leptospiral load in organs infected with *L. weilii* strain SC295 and *L. interrogans* strain HP358.

*Leptospira* species	Blood	Lungs	Liver	Kidneys
***L. weilii* stra5in SC295**				
S1	NA	NA	NA	1.326 × 10^3^
S2	NA	NA	NA	2.740 × 10^5^
S3	NA	NA	NA	1.159 × 10^3^
S4	NA	NA	NA	1.67 × 10^2^
**Average**	**NA**	**NA**	**NA**	**6.916 × 10^4^**
***L. interrogans* strain HP358**				
S1 (died at D6)	NA	6.390 × 10^6^	5.240 × 10^8^	7.450 × 10^7^
S2 (died at D6)	NA	4.290 × 10^6^	1.330 × 10^8^	2.750 × 10^7^
S3 (died at D7)	NA	1.988 × 10^4^	1.270 × 10^6^	9.620 × 10^6^
S4 (euthanized at D7)	2.018 × 10^3^	3.134 × 10^4^	6.585 × 10^4^	2.460 × 10^7^
**Average**	**2.018 × 10^3^**	**2.683 × 10^6^**	**1.646 × 10^8^**	**3.405 × 10^7^**

*Average values are indicated in bold.*

### General Genomic Descriptions

**FIGURE 4:**
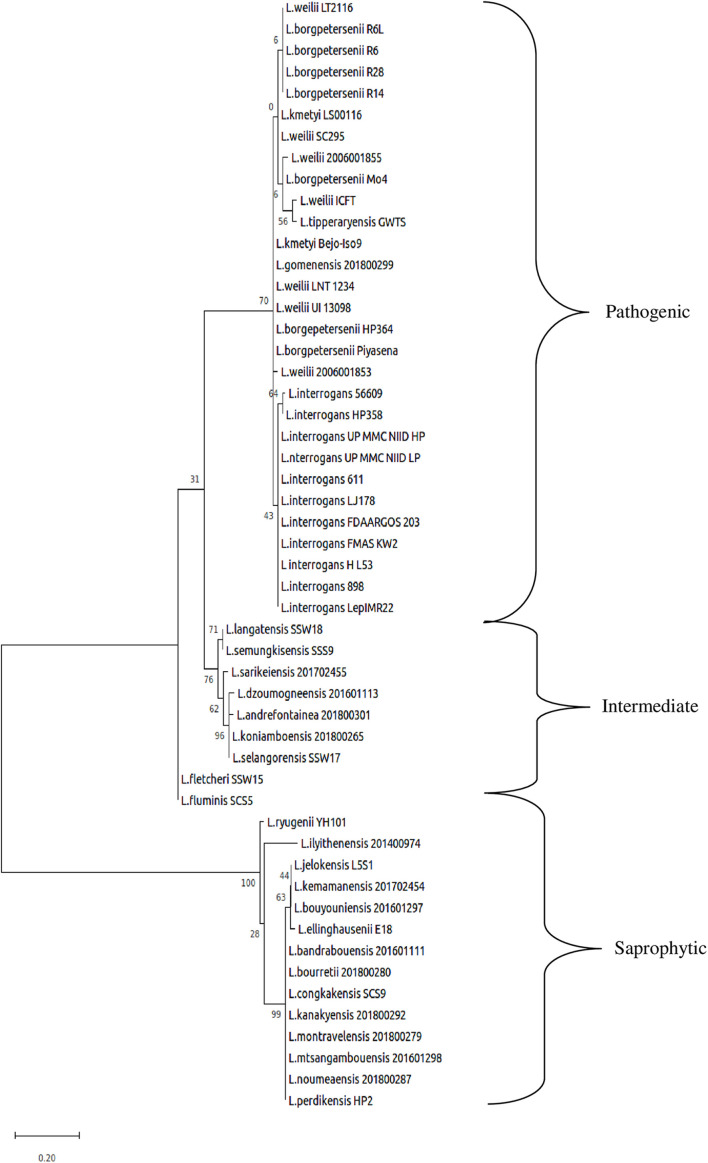
Whole-genome core single-nucleotide polymorphisms (core SNPs) phylogeny of *Leptospira* isolates based on the maximum likelihood method with 1,000 bootstrap replicates.

**FIGURE 5:**
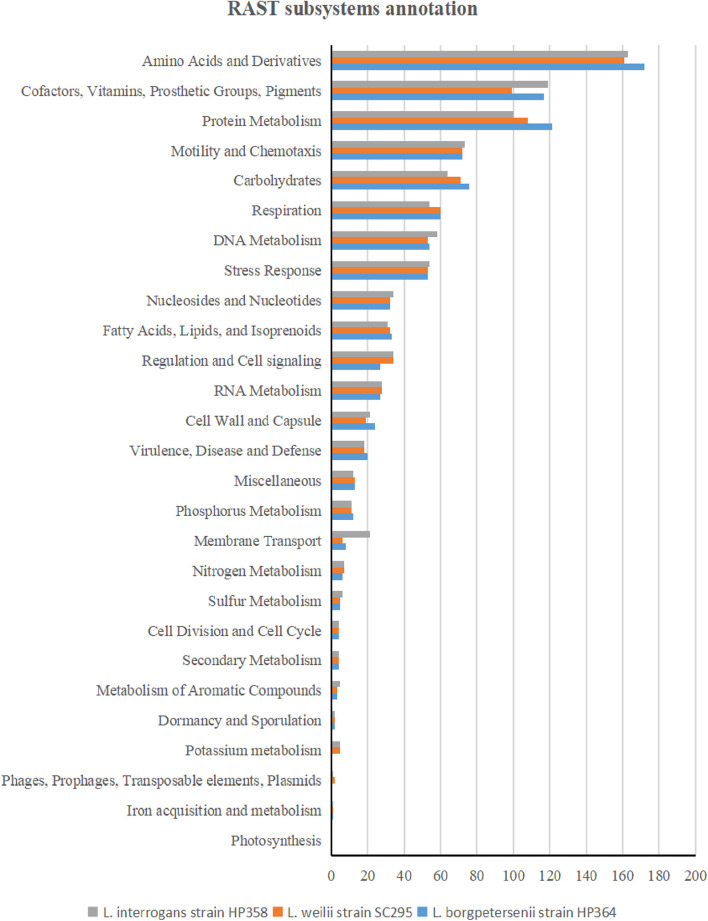
RAST subsystem annotation in *L. borgpetersenii* strain HP364, *L. weilii* strain SC295, and *L. interrogans* strain HP358.

The whole-genome size of *L. borgpetersenii* strain HP364, *L. weilii* strain SC295, and *L. interrogans* strain HP358 was determined to be 3,904,517; 4,111,826; and 4,808,724 bp with GC content (40.1, 40.2, and 35.0%) and predicted gene and protein-coding sequences (4,323; 4,691; and 4,733), respectively. The genetic variations based on SNP (Figure 4) confirm that *L. borgpetersenii* strain HP364, *L. weilii* strain SC295, and *L. interrogans* strain HP358 are pathogenic species and clustered among its respective species as established earlier (Azhari et al., 2018; Vincent et al., 2019). The low support value on the separation between the pathogenic and intermediate groups might be due to low samples size, and SNP is based on the genotype analysis. RAST categorized the functions of the coding sequences (CDSs) in strains HP364, SC295, and HP358 into 661 (16%), 648 (15%), and 661 (13%) subsystem coverage containing 946, 903, and 930 CDSs, respectively (Supplementary File 2). Most CDSs in strains HP364, SC295, and HP358 related to amino acids and derivatives (172/946, 161/903, 163/930), cofactors, vitamins, prosthetic groups, pigments (117/946, 99/903, 119/930), protein metabolism (121/946, 108/903, 100/930), motility and chemotaxis (72/946, 72/903, 73/930), and carbohydrates (76/946, 71/903, 64/930) (Figure 5).

**FIGURE 6:**
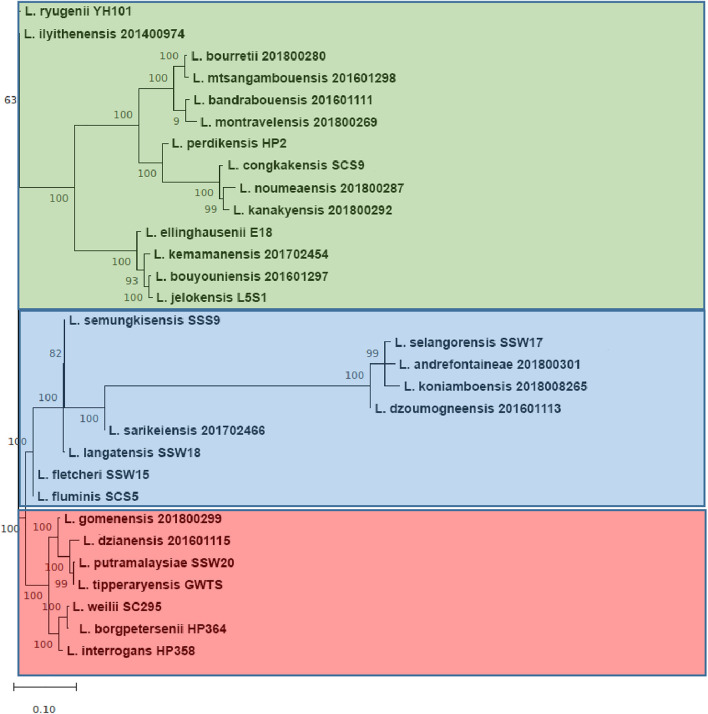
The core-genome phylogenetic tree. The group colored red, blue, and green corresponding to pathogenic, intermediate, and non-pathogenic clades, respectively. Each major branch of tree was supported with very high bootstrap 1,000.

**FIGURE 7:**
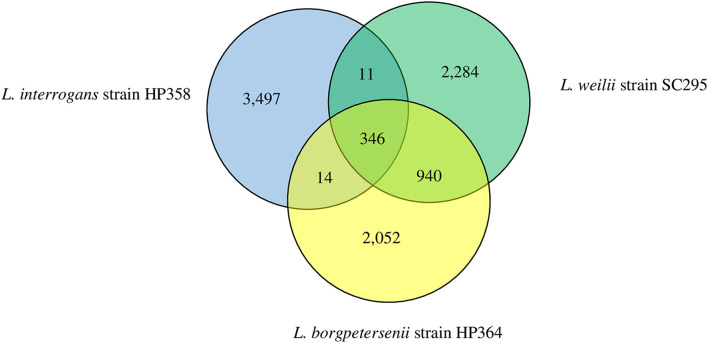
Pan-genome overview. The Venn diagram presents the core genome and the number of genes specific for *L. borgpetersenii* strain HP364, *L. weilii* strain SC295, and *L. interrogans* strain HP358.

The range of pan-genome size for all the 30 *Leptospira* strains used in the pan-genome analysis is 3,352–4,658 (Supplementary File 3, Tables 1, 2). The phylogenetic tree showing the clustering of the strains is shown in Figure 6. The pan-genome analysis performed on three pathogenic *Leptospira* species presented genome sizes of 3,352; 3,581; and 3,868 for *L. borgpetersenii* strain HP364, *L. weilii* strain SC295, and *L. interrogans* strain HP358. From a total 9,144 genes, 346 were present in the core genome with 95% sequence identity among the species (Figure 7). A list of gene presence and absence from the pan-genome analysis in these three pathogenic species is shown in Supplementary File 3, Table 3.

### Presence of Known Virulence Factors

The basic genome characteristics of *Leptospira* species and strains used in known virulence analysis are summarized in Supplementary File 4. Among the 38 *Leptospira*-associated virulence factors, Loa22, FliN, ClpB, HtpG, LipL32, hemolysin B, and hemolysin C were found to be common among the pathogenic *Leptospira* species and strains with ≥ 90% identity (Supplementary File 5, Table 1). Two (leptospiral endostation-like protein D and thermolysin) and one (leptospiral endostation-like protein D) virulence factors in *L. borgpetersenii* strain HP364 and *L. weilii* strain SC295 were found to be less than 40% identity with the *L. interrogans* species and strains (Table 5). Gene *Lsa21* was only detected among the *L. interrogans* strains. All virulence factors except for LigA-like protein and leptospiral endostatin-like protein F in *L. interrogans* strain HP358 revealed 97–100% similarity with the other *L. interrogans* strains. In intermediate and saprophytic *Leptospira* species, all virulence factors except hemolysin B have ≤ 80% identity and the alignment hit for *vwa-I*, *vwa-II*, *Lsa21*, and *invA* are not significant. Collagenase, putative lipoprotein (LB194), hypothetical protein (LA2786), LigA-like protein, LipL32, and sphingomyelinase C are not found in some of the intermediate and saprophytic species.

**TABLE 5:** The presence/absence and percentage of homology of the known virulence factors in each tested strain.

Protein/Gene	HP364	SC295	HP 358	SSW18	SSS9	SSW17	SSW15	SCS9	L5S1	HP2
HO	88	87	99	55	56	56	27	44	44	45
Loa22	92	93	98	69	70	66	67	53	55	52
FliN	90	90	100	73	73	74	74	56	56	56
ClpB	90	91	99	77	77	77	76	43	43	42
FlaA2	90	88	100	73	74	73	74	57	58	58
Catalase	87	87	100	30	55	26	28	75	27	75
MCE	89	89	100	73	72	73	73	60	61	61
LipL71	85	87	100	63	62	62	59	38	37	37
Collagenase	85	76	99	39	31	28	34	38	34	32
HbpA	84	85	99	54	54	55	54	30	31	30
HtpG	92	93	99	72	73	74	74	64	65	65
Putative lipoprotein (LB194)	76	76	99	NF	36	31	28	34	34	NF
Hypothetical protein (LA2786)	48	48	98	41	35	NF	27	30	26	27
Hypothetical protein (LA0589)	61	61	99	36	47	34	26	24	39	26
LigA	53	54	87	41	41	42	30	NF	33	NF
LigB	64	65	97	47	47	48	39	37	35	25
LigC	87	89	99	63	63	63	29	24	45	24
LipL32	98	95	100	68	69	66	70	28	37	NF
OmpL1	85	86	100	47	47	48	51	46	45	45
LipL45	89	88	99	62	62	62	67	56	55	55
Tly A	81	83	99	52	53	54	52	47	47	47
Tly B	96	96	99	87	87	87	87	78	78	79
Tly C	94	94	100	72	73	72	73	57	58	57
sphH	54	55	99	27	38	32	27	33	40	26
sph2	58	68	99	40	30	29	42	50	32	50
Len A	65	63	98	34	31	29	37	36	30	30
Len B	53	52	100	28	33	28	NS	35	31	48
Len C	40	61	99	27	35	32	34	34	31	36
Len D	35	32	99	31	29	40	37	35	29	39
Len E	44	37	100	31	29	31	40	45	44	44
Len F	55	72	63	31	34	33	35	39	32	38
Thermolysin	30	62	100	44	44	30	23	26	33	25
Peroxidase	83	80	99	67	68	69	38	51	49	51
*vwa-I*	85	80	99	NS	NS	NS	NS	NS	NS	NS
*vwa-II*	80	80	99	NS	NS	NS	NS	NS	NS	NS
*Lsa21*	NS	NS	99	NS	NS	NS	NS	NS	NS	NS
*invA*	82	82	99	NS	NS	NS	81	NS	NS	NS
*lipL21*	89	90	100	81	80	80	82	NS	NS	NS

### Potential Putative Virulence Factors

From VFDB database, the genomes of *L. borgpetersenii* strain HP364, *L. weilii* strain SC295, and *L. interrogans* strain HP358 were shown to encode 109, 109, and 110 putative virulence factors, respectively (Supplementary File 5, Tables 2–4). Victors database analysis provided a higher number of predictive virulence factors, with 165, 166, and 161 in *L. borgpetersenii* strain HP364, *L. weilii* strain SC295, and *L. interrogans* strain HP358, respectively (Supplementary File 5, Tables 5–7). Among all the predicted virulence factors, 65 (VFDB) and 128 (Victors) are common in the three *Leptospira* species (Supplementary File 5, Tables 8–9). The predicted virulence factors in VFDB databases are involved in adherence, metabolic adaptation, endotoxin, invasion, motility, stress protein, immune evasion, antiphagocytosis, secretion system, enzyme, regulation, amino acid, and purine metabolism, colonization, biofilm formation, phase variation, intracellular survival and entry, immunomodulator, structural mimicry, serum resistance, and toxin. The common predictive virulence factors shared between *L. borgpetersenii* strain HP364 and *L. weilii* strain SC295 (VFDB: 20, Victors: 18) are higher than in *L. borgpetersenii* strain HP364/*L. interrogans* strain HP358 (VFDB and Victors: 8) and *L. weilii* strain SC295/*L. interrogans* strain HP358 (VFDB: 8, Victors: 10) (Supplementary File 5, Tables 10–12). Both VFDB and Victors databases revealed the highest number of specific putative virulence factors in *L. interrogans* strain HP358 genome (VFDB: 29, Victors: 15) (Supplementary File 5, Table 13). In addition to the aforementioned roles/functions, one specific putative virulence factor, sphingomyelinase C (SMase) in *L. interrogans* HP358, is involved in exoenzyme or membrane damaging. In *L. borgpetersenii* strain HP364 and *L. weilii* strain SC295, the numbers of specific putative virulence factors are 16, 16 (VFDB) and 11, 10 (Victors) (Supplementary File 5, Tables 14, 15), respectively. From the pan-genome analysis, a list of genes that were characterized as virulence factors in other bacterial species was identified in *L. interrogans* HP358 genome. These genes included Lon protease, E3 ubiquitin–protein ligase sspH1, colicin I receptor (*cirA*), and *gluP* endotoxin (Rhomboid protease GluP) (Supplementary File 3, Table 3).

## Discussion

The diversity of *Leptospira* species, serogroups, and serovars contributes to the wide variety of clinical presentations of leptospirosis. In this study, we investigated the *in vivo* (hamster model) virulence potential of *Leptospira* species isolated from the environments and rodents captured from the leptospirosis outbreak areas in Malaysia. None of the *Leptospira* species isolated from environments induced illness in hamsters, confirming the molecular characterization of these species as non-pathogens. The three pathogenic *Leptospira* species showed distinct virulence phenotype characteristics. Although *L. borgpetersenii* belongs to the pathogenic group of *Leptospira*, strain HP364 did not induce any clinical signs in hamsters. Reports from studies elsewhere elucidated mixed pathogenic potentials. *L. borgpetersenii* strain 2014TM FMNH228 isolated from bat (*Triaenops menamena*) in Madagascar did not induce any clinical signs in hamsters (Cordonin et al., 2019). Two strains of *L. borgpetersenii* serovar Hardjo, JB197 and 203 isolated from steers in the United States, were able to successfully infect hamsters but differed widely in clinical outcomes; the strain JB197 developed rapidly debilitating disease with a fatal outcome, whereas with the strain 203, although it established chronic renal infections, the hamsters remained asymptomatic (Miller et al., 1991; Zuerner et al., 2012). In other studies, *L. borgpetersenii* serovar Javanica strain K6 (isolated from rats in the Philippines) and Ballum strain 4E (isolated from *Mus musculus* in Brazil) induced clinical presentations typical of acute leptospirosis (Silva et al., 2010; Diniz et al., 2011; Villanueva et al., 2014). For hamsters infected with *L. weilii* strain SC295, animals developed mild symptoms, although damages in the kidney were observed. Similar findings were also reported from China, where *L. weilii* serovar Heyan (isolated from a patient) exhibited low virulence in guinea pig animal model (Xu et al., 2017).

*L. interrogans* strain HP358 induced severe illness in hamsters, causing fatality as early as day 6 p.i. This severe presentation was characterized as sudden death in some animals after the onset of clinical presentations. In previous studies performed in hamsters or guinea pig infected with different serovars/strains of *L. interrogans*, the strain 2013RR GLM983 isolated from *Rattus rattus* in Western Indian Ocean (Cordonin et al., 2019), serovar Manilae strain K64, Losbanos strain K37 and Ratnapura strain K5 isolated from rats in the Philippines (Villanueva et al., 2014), serovar Icterohaemorrhagiae strain Verdun (Reference Collection of the Institut Pasteur in Paris, France) (Matsui et al., 2011), serovar Icterohaemorrhagiae strain no 1143 (FIOCRUZ laboratory, Brazil) (Marinho et al., 2009), serovar Copenhageni strains RJ15958 and RJ1644 isolated from patients with severe pulmonary form of leptospirosis in Brazil (Nally et al., 2004), serovar Manilae strain UPMMC isolated from human patients with severe leptospirosis in the Philippines (Koizumi and Watanabe, 2003), and serotype Icterohaemorrhagiae strain NADL-H14 (van den Ingh and Hartman, 1986) developed acute leptospirosis and resulted in fatal outcomes. In general, hemorrhages associated with fatality were the main clinical presentations in the hosts infected with *L. interrogans* in most of the studies.

It was observed from this study that the presence of leptospires in organs contributes to the clinical consequences in host cells. As shown in Table 4, there were differences in the leptospiral colonization and number in animals infected with *L. weilii* strain SC295 and *L. interrogans* strain HP358. Leptospires were detected only in the kidney infected with *L. weilii* strain SC295, which was in accordance with the pathological changes observed in the kidney and none in the lungs and liver. In hamsters infected with *L. interrogans* strain HP358, leptospires were detected in all organs (lungs, liver, and kidneys), in accordance with the pathological changes in these organs. There were no leptospires detected in animals infected with *L. borgpetersenii* strain HP364, and thus, no pathological changes were observed in the lungs, liver, and kidneys. Hence, it can be concluded that besides the exacerbated cytokines responses (Cagliero et al., 2018; Senavirathna et al., 2020), the leptospiral dynamics also contribute to the clinical consequences in the host cells as reported in previous studies (Miyahara et al., 2014; Xu et al., 2020).

The differences in the virulence level among the species could be attributed to the wide variety of genes contained in the genome of each *Leptospira* isolates. The identification and characterization of virulence factors are important for understanding leptospiral pathogenesis, interaction with the host, and developing diagnostics, vaccines, and new drugs. Through RAST analysis, the genomes of the *L. borgpetersenii* strain HP364, *L. weilii* strain SC295, and *L. interrogans* strain HP358 were found to have approximately similar numbers of CDs and subsystems, which include amino acids and derivatives, cofactors, vitamins, prosthetic groups, pigments, protein metabolism, motility and chemotaxis, and carbohydrates. However, as subsystems coverage in RAST is only between 13 and 16% from the whole genomes and as revealed from the pan-genome analysis that these three species share only 346 core genes, there are many more genes that need to be explored and elucidated to understand the determinants of virulence characteristics. Indeed, from the known virulence factor analysis, the nucleotide sequence identity in the genome of *L. interrogans* strain HP358 differs from *L. borgpetersenii* strain HP364 and *L. weilii* strain SC295. Leptospiral endostation-like protein D and thermolysin, which are involved in adherence and invasion of *Leptospira*, showed less than 40% identity in *L. borgpetersenii* strain HP364 and *L. weilii* strain SC295. *L. interrogans* strain HP358 encodes the *Lsa21* gene (absent in *L. borgpetersenii* strain HP364 and *L. weilii* strain SC295) and had been reported to induce strong production of proinflammatory cytokines *via* TLR2 and TLR4 signaling in mouse macrophage (Faisal et al., 2016), and this might also explain the high virulence capability of *L. interrogans* strain HP358. As expected, most of the known virulence factors showed less than 80% identity in the genomes of intermediate and saprophytic species. Overall, the absence or low similarity of virulence factors among the pathogenic *Leptospira* species may explain the difference in their capability to cause disease in infected hosts.

From the VFDB and Victors databases, approximately 109–110 and 161–166 genes in the genome of *L. borgpetersenii* strain HP364, *L. weilii* strain SC295, and *L. interrogans* strain HP358 were predicted to share orthologs with the virulence factors in other bacteria species. *L. interrogans* strain HP358 revealed a higher number of specific putative virulence factors compared to the other two *Leptospira* species. Besides VFDB and Victors analyses, the absence and presence of genes in pan-genome analysis also revealed that the genome of *L. interrogans* strain HP358 encodes several genes that are known to be virulence factors in other bacterial species such as Lon protease. The Lon mutants *Salmonella*, C*ampylobacter*, and *Pseudomonas aeruginosa* showed less severe infection in various animal models (Takaya et al., 2003; Boddicker and Jones, 2004; Brazas et al., 2007; Cohn et al., 2007; Marr et al., 2007; Breidenstein et al., 2008). Another potential virulence factor is *cirA*, a gene that encodes the colicin I receptor. *CirA* mutant *Salmonella enteritidis* strain C50336 showed a sharp decrease in biofilm formation and impaired antibiotic resistance (Zhang et al., 2020). The genome of *L. interrogans* strain HP358 also contains sspH1 gene that encodes the E3 ubiquitin–protein ligase SspH1. This protein (also known as effector protein) has been reported to alter the host cell physiology and promote bacterial survival by interfering with the host’s ubiquitination pathway and target the host proteins for proteasomal degradation (Pisano et al., 2018). *L. interrogans* strain HP358 also encodes the gene *gluP*, which has been reported as an endotoxin in *Helicobacter* (Saeb et al., 2014). However, to understand the expression and function of these known virulence genes of other organisms in *Leptospira*, mutant studies, functional analysis, and expression studies are vital.

*L. interrogans* strain HP358 induced hemorrhage in hamster model, typical infection by most *L. interrogans* serovars/strain. Indeed, patients with severe leptospirosis reported in Malaysia had hemorrhagic manifestations. Leptospirosis with pulmonary hemorrhage was observed in patients and returned travelers from Malaysia (Wagenaar et al., 2004; Leung et al., 2011; Nor et al., 2016; Lim et al., 2018). Although the infecting *Leptospira* species or serovars were not known except for one case caused by serovar Lai: Langkawi (Wagenaar et al., 2004), it showed that the leptospires circulating in Malaysia have the capability to cause severe disease with hemorrhagic manifestations. The known virulence factors in the genome of *L. interrogans* strain HP358 also share a similar identity with the other two *L. interrogans* isolates in Malaysia (*L. interrogans* serovar Batavia strain LepIMR 22 and *L. interrogans* serovar Icterohaemorrhagiae strain 898). Therefore, it is imperative to perform a detailed study on *L. interrogans* strains isolated in Malaysia to understand the extent of damages to different organs and the inflammatory response to effectively understand the prognosis of the illness and efficient management.

## Conclusion

In conclusion, *Leptospira* species plays an important role in determining the wide clinical presentation of leptospirosis. The new *L. interrogans* strain HP358 isolate showed a high level of virulence. This strain not only harbors the known virulence genes of *Leptospira* but also encodes genes known to be virulence factors in other bacteria. Further studies are recommended to confirm the role of these potential virulence factors in the virulence of this strain and in other *Leptospira* species.

## Data Availability Statement

The datasets presented in this study can be found in online repositories. The names of the repository/repositories and accession number(s) can be found in the article/Supplementary Material.

## Ethics Statement

The animal study was reviewed and approved by the Institutional Animal Care and Use Committee (IACUC), Universiti Putra Malaysia with Animal Use Protocol (AUP) number: UPM/IACUC/AUP-R044/2018.

## Author Contributions

NP performed the study, interpreted the data, and drafted the manuscript. JJ performed the bioinformatic analysis and wrote that part of the manuscript. NA collected the *Leptospira* isolates. ZS helped with study design. VKN designed and supervised the study and reviewed and finalized the manuscript. All authors read and approved the manuscript.

## Conflict of Interest

The authors declare that the research was conducted in the absence of any commercial or financial relationships that could be construed as a potential conflict of interest.

## Publisher’s Note

All claims expressed in this article are solely those of the authors and do not necessarily represent those of their affiliated organizations, or those of the publisher, the editors and the reviewers. Any product that may be evaluated in this article, or claim that may be made by its manufacturer, is not guaranteed or endorsed by the publisher.

## Footnotes

1
https://www.blaststation.com/intl/en/blaststation2.php

2
http://www.phidias.us/victors/download.php
