# Supplementary material for: In vivo and in silico Virulence Analysis of Leptospira Species Isolated From Environments and Rodents in Leptospirosis Outbreak Areas in Malaysia
Source: Front Microbiol. 2021 Nov 5;12:753328. doi: 10.3389/fmicb.2021.753328 (PMC8602918; doi:10.3389/fmicb.2021.753328)
Supplement: Supplementary file 4 [file Table_4.DOC]

| **No.** | **Species**  **Supplementary File 4**: Representative *Leptospira* species and strains used in known virulence analysis. | **Strain** | **Serovars** | **Species of origin** | **Genome status** | **Origin of isolate** | **Genome size**  **(bp)** | **GC%** | **Accession Number** |
| --- | --- | --- | --- | --- | --- | --- | --- | --- | --- |
| 1. | *L. borgpetersenii* | HP364 | NA | Rodent | Draft | Malaysia | 3,904,517 | 40.1 | NA |
| 2. | *L. borgpetersenii* | Piyasena | Ceylonica | NA | Complete | NA | 3,992,782 | 40.1 | CP026672.1 |
| 3. | *L. borgpetersenii* | R14 | NA | Rodent | Chromosome | Caribbean | 3,908,796 | 40.0 | CP047504.1 |
| 4. | *L. borgpetersenii* | R28 | NA | Rodent | Chromosome | Caribbean | 3,887,691 | 40.0 | CP047332.1 |
| 5. | *L. borgpetersenii* | R6 | NA | Rodent | Chromosome | Caribbean | 3,821,116 | 40.0 | CP047372.1 |
| 6. | *L. borgpetersenii* | R6L | NA | Rodent | Chromosome | Caribbean | 3,887,609 | 40.0 | CP047520.1 |
| 7. | *L. borgpetersenii* | Mo4 | NA | Rodent | Chromosome | Caribbean | 3,888,971 | 40.0 | CP047334.1 |
| 8. | *L. borgpetersenii* | 203 | Hardjo-bovis | Cattle | Complete | USA | 3,907,328 | 40.2 | CP021412.1 |
| 9. | *L. borgpetersenii* | JB197 | Hardjo-bovis | NA | Complete | NA | 3,876,235 | 40.2 | CP000350.1 |
| 10. | *L. borgpetersenii* | 4E | Ballum | Rodents | Chromosome | Brazil | 3,912,599 | 40.2 | CP015814.2 |
| 11. | *L. weilii* | SC295 | NA | Rodent | Draft | Malaysia | 4,111,826 | 40.2 | NA |
| 12. | *L. weilii* | CUDO6 | NA | Dog | Complete | Thailand | 4,381,393 | 40.9 | NZ_CP040840.1 |
| 13. | *L. weilii* | L231 | Heyan | Human | Contig | China | 4,265,366 | 40.7 | MSFX01000001.1 |
| 14. | *L. weilii* | CUD13 | NA | Dog | Complete | Thailand | 4,361,439 | 40.9 | CP040843.1 |
| 15. | *L. interrogans* | HP358 | NA | Rodent | Draft | Malaysia | 4,808,724 | 35.0 | JAFCYY000000000.1 |
| 16. | *L. interrogans* | LepIMR 22 | Bataviae | Rodent | Contig | Malaysia | 4,723,435 | 35.2 | LUVO01000001.1 |
| 17. | *L. interrogans* | 898 | Icterohaemorrhagiae | Human | Complete | Malaysia | 4,630,592 | 35.0 | CP043891.1 |
| 18. | *L. interrogans* | Verdun LP | Icterohaemorrhagiae | Human | Contig | France | 4,609,830 | 35.0 | AKWP02000031.1 |
| 19. | *L. interrogans* | 56609 | Linhai | NA | Complete | China | 4,915,652 | 35.0 | CP006723.1 |
| 20. | *L. interrogans* | UP-MMC-NIID HP | Manilae | Rodent | Complete | Philippine | 4,667,354 | 35.0 | CP011934.1 |
| 21. | *L. interrogans* | UP-MMC-NIID LP | Manilae | Rodent | Complete | Philippine | 4,667,405 | 35.0 | CP011931.1 |
| 22. | *L. interrogans* | 611 | Canicola | Human | Complete | China | 4,755,342 | 35.0 | CP044513.1 |
| 23. | *L. interrogans* | LJ178 | Canicola | NA | Complete | China | 4,758,488 | 35.0 | CP044509.1 |
| 24. | *L. interrogans* | FMAS_KW2 | NA | Human | Complete | Sri Lanka | 4,651,137 | 35.0 | CP039256.1 |
| 25. | *L. interrogans* | FDAARGOS_203 | Copenhageni | Human | Complete | Brazil | 4,630,763 | 35.0 | CP020414.2 |
| 26. | *L. interrogans* | SK1 | Copenhageni | Dog | Complete | Caribbean | 4,630,180 | 35.0 | CP048830.1 |
| 27. | *L. interrogans* | Andaman | Grippotyphosa | NA | Contig | NA | 4,937,420 | 35.1 | AKXG02000001.1 |
| 28. | *L. interrogans* | 2006006986 | Grippotyphosa | Human | Complete | [Egypt](https://www.ncbi.nlm.nih.gov/biosample?term=) | 4,935,986 | 35.5 | NZ_AKXC02000057.1 |
| 29. | *L. interrogans* | 2006006971 | Grippotyphosa | NA | Scaffold | NA | 4,805,969 | 35.0 | AFJO01000001.1 |
| 30. | *L. interrogans* | 56601 | Lai | NA | Complete | China | 4,698,134 | 35.0 | AE010300.2 |
| 31. | *L. interrogans* | AKRFB | Pomona | Cattle | Scaffold | Argentina | 4,627,117 | 35.0 | NZ_LUHH01000031.1 |
| 32. | *L. langatensis* | SSW18 | NA | Water | Scaffold | Malaysia | 4,089,386 | 44.8 | NZ_RQER01000004.1 |
| 33. | *L. semungkisensis* | SSS9 | NA | Soil | Contig | Malaysia | 3,944,076 | 42.8 | NZ_RQEP01000005.1 |
| 34. | *L. selangorensis* | SSW17 | NA | Water | Contig | Malaysia | 4,191,189 | 40.0 | NZ_RQES01000005.1 |
| 35. | *L. fletcheri* | SSW15 | NA | Water | Contig | Malaysia | 3,733,663 | 47.3 | NZ_RQET01000004.1 |
| 36. | *L. congkakensis* | SCS9 | NA | Soil | Contig | Malaysia | 4,003,841 | 38.2 | NZ_RQGQ01000004.1 |
| 37. | *L. jelokensis* | L5S1 | NA | Soil | Contig | Malaysia | 4,124,627 | 38.9 | NZ_RQGR01000029.1 |
| 38. | *L. perdikensis* | HP2 | NA | Water | Contig | Malaysia | 4,001,979 | 38.5 | NZ_RQGA01000003.1 |
|  | *L. biflexa* | Patoc 1 | Patoc | NA | Complete | NA | 4,627,117 | 38.9 | CP000786.1 |
